# Toll-like receptor 9 negatively regulates pancreatic islet beta cell growth and function in a mouse model of type 1 diabetes

**DOI:** 10.1007/s00125-018-4705-0

**Published:** 2018-08-09

**Authors:** Mengju Liu, Jian Peng, Ningwen Tai, James A. Pearson, Changyun Hu, Junhua Guo, Lin Hou, Hongyu Zhao, F. Susan Wong, Li Wen

**Affiliations:** 1 Section of Endocrinology, Department of Internal Medicine, Yale School of Medicine, Yale University, 300 Cedar Street, New Haven, CT 06520 USA; 2 Present Address: Jounce Therapeutics Inc., Cambridge, MA USA; 3 Present Address: Department of Rheumatology, PLA General Hospital, Beijing, People’s Republic of China; 4 Department of Bioinformatics, Yale School of Public Health, Yale University, New Haven, CT USA; 5 Division of Infection and Immunity, Cardiff University School of Medicine, Cardiff, CF14 4XN UK

**Keywords:** CD140a, Diabetes, Islet beta cell, PDGFRα, TLR9

## Abstract

**Aims/hypothesis:**

Innate immune effectors interact with the environment to contribute to the pathogenesis of the autoimmune disease, type 1 diabetes. Although recent studies have suggested that innate immune Toll-like receptors (TLRs) are involved in tissue development, little is known about the role of TLRs in tissue development, compared with autoimmunity. We aimed to fill the knowledge gap by investigating the role of TLR9 in the development and function of islet beta cells in type 1 diabetes, using NOD mice.

**Methods:**

We generated *Tlr9*^−/−^ NOD mice and examined them for type 1 diabetes development and beta cell function, including insulin secretion and glucose tolerance. We assessed islet and beta cell number and characterised CD140a expression on beta cells by flow cytometry. We also tested beta cell function in *Tlr9*^−/−^ C57BL/6 mice. Finally, we used TLR9 antagonists to block TLR9 signalling in wild-type NOD mice to verify the role of TLR9 in beta cell development and function.

**Results:**

TLR9 deficiency promoted pancreatic islet development and beta cell differentiation, leading to enhanced glucose tolerance, improved insulin sensitivity and enhanced first-phase insulin secretory response. This was, in part, mediated by upregulation of CD140a (also known as platelet-derived growth factor receptor-α [PDGFRα]). In the absence of TLR9, induced by either genetic targeting or treatment with TLR9 antagonists, which had similar effects on ontogenesis and function of beta cells, NOD mice were protected from diabetes.

**Conclusions/interpretation:**

Our study links TLR9 and the CD140a pathway in regulating islet beta cell development and function and indicates a potential therapeutic target for diabetes prevention and/or treatment.

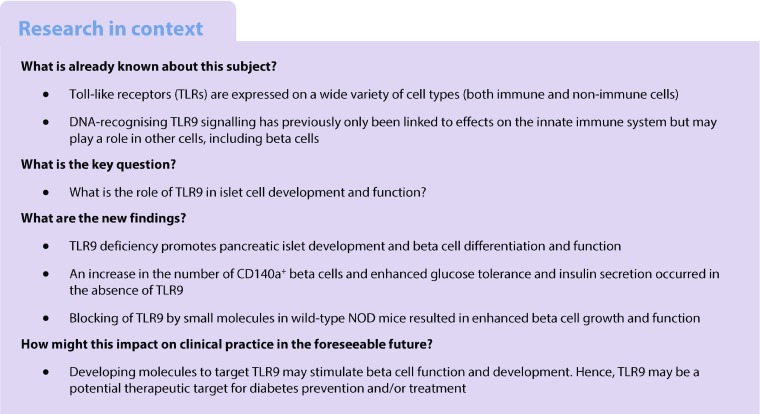



## Introduction

The innate immune system generates early inflammatory responses to a variety of environmental insults. A large number of innate immune receptors, including the Toll-like receptors (TLRs), are important for immediate immune responses to infection, leading to later, more specific, adaptive immunity. On binding the appropriate ligand, the TLRs activate signalling pathways that lead to production of proinflammatory cytokines and upregulation of costimulatory molecules. TLRs were initially thought to be expressed mainly on immune cells, in particular antigen-presenting cells, but it is increasingly recognised that they are also expressed on many other cell types and have functions that range beyond activation of the immune system. We, and others, have shown that pancreatic beta cells express many TLRs in both mice and humans [1, 2]. Activation of TLR3, a receptor for double-stranded RNA, has been shown to induce beta cell apoptosis [1, 3, 4]. TLR4, the receptor for endotoxin, is involved in regulation of metabolism in a variety of tissues including beta cells [5–7]. TLR9 can also be detected easily in both mouse and human islets [1, 2].

Type 1 diabetes is a slowly progressing autoimmune disease. We, and others, have independently shown that TLR9-deficient (*Tlr9*^−/−^) NOD mice are protected from type 1 diabetes development [8–10]. This protection is mediated partly by impaired IFNα production from *Tlr9*^−/−^ mouse dendritic cells [9] and by enhanced expression and regulatory function of CD73^+^ T cells [10]. However, increasing evidence suggests that TLRs recognise not only exogenous ligands from microbes but also endogenous ligands from both normal and damaged cells. Recent studies suggest that DNA released from both physiological and pathological dying cells can be a key stimulus to innate immune activation of TLR9 [11–14]. There is also evidence that TLRs regulate neurogenesis during development [15]. Considering that islet beta cells undergo significant growth and remodelling, early in life [16–19], it is likely that TLR9 plays an important role in the development of type 1 diabetes, beyond any direct immune function. However, to date, there have been no reports about the role of TLR9 in islet beta cell development. Therefore, we aimed to assess the role of TLR9 in the development and function of islet beta cells in both NOD and C57BL/6 mice.

## Methods

### Mice

All the mice used in the study were housed in specific pathogen-free conditions with a 12 h dark–light cycle and were housed in individually ventilated filter cages with autoclaved food and bedding at the Yale University animal facility. The *Tlr9*^−/−^ NOD mice were generated by backcrossing *Tlr9*^−/−^ C57BL/6 mice [20] with our NOD mice, for over 11 generations. The purity of the NOD genetic background was confirmed by mouse genome SNP scan with Illumina Infinium panel (DartMouse, Lebanon, NH, USA). *Tlr9*^−/−^ NOD.Scid mice were generated by breeding *Tlr9*^−/−^ NOD mice with NOD.Scid mice, which were originally purchased from the Jackson Laboratory (Bar Harbor, ME, USA) and maintained at Yale University for ~25 years. Wild-type (WT) C57BL/6 (*Tlr9*^+/+^ C57BL/6) mice were also purchased from the Jackson Laboratory and maintained at Yale University for ~10 years. The use of the animals in this study was approved by the IACUC of Yale University. All mice used in different experiments were randomly selected from different breeding cages and different litters. Experimenters were not blinded in this study.

### Natural history of diabetes development

*Tlr9*^−/−^ NOD mice and *Tlr9*^+/+^ NOD littermates were screened for glycosuria weekly for spontaneous diabetes development, up to 32 weeks of age. Diabetes was confirmed by blood glucose of ≥13.9 mmol/l with a FreeStyle glucose meter (Abbott, Chicago, IL, USA).

### Streptozotocin-induced diabetes development

Female *Tlr9*^−/−^ NOD mice and *Tlr9*^+/+^ NOD littermates (5–6 weeks old) were treated with either high-dose streptozotocin (STZ) (100 mg/kg, administered by two consecutive i.p. injections, 24 h apart) or low-dose STZ (40 mg/kg, administered by i.p. injection, once daily, for 5 days). Mice were screened for glycosuria daily for diabetes development and confirmed as above.

### Intra-peritoneal glucose tolerance test

Intra-peritoneal glucose tolerance tests (IPGTTs) were performed in 5–6-week-old *Tlr9*^−/−^ NOD, *Tlr9*^+/+^ NOD, *Tlr9*^−/−^ C57BL/6, *Tlr9*^+/+^ C57BL/6, *Tlr9*^−/−^ NOD.Scid and *Tlr9*^+/+^ NOD.Scid mice. The mice were fasted overnight with free access to water and the blood glucose was measured before (time zero) and after i.p. injection of glucose (1 g/kg) at different time points from blood samples. Blood glucose was measured by a FreeStyle glucose meter (Abbott). Data are shown from one out of three experiments, each confirming the significant difference.

### Insulin tolerance test

Insulin tolerance tests (ITTs) were performed in 5–6-week-old male *Tlr9*^−/−^ C57BL/6 mice and *Tlr9*^+/+^ C57BL/6 mice. The mice were fasted for 6 h with free access to water and the blood glucose was measured before and after i.p. injection of insulin (Humulin-R, 0.75 U/kg; Eli Lilly, Indianapolis, IN, USA) at different time points, as described for IPGTT.

### Islet and beta cell isolation

Pancreatic islets were isolated as previously described [21]. Mice were euthanised by cervical dislocation. The pancreas was inflated with 3 ml cold collagenase (Sigma; St Louis, MO, USA) solution (0.3 mg/ml) through the bile duct with a 20G needle starting at the gall bladder. The pancreas was then removed into a siliconised glass tube containing 2 ml of 1 mg/ml collagenase solution and digested at 37°C in a water bath for 12–15 min. After three washes of the digested pancreas, islets were hand-picked and counted under a dissecting microscope for further experiments. For single-cell isolation, the islets were treated with Cell Dissociation Solution (Sigma) and the single-cell suspension was harvested. Beta cells from the dissociated islets were stained with fluorochrome-conjugated monoclonal antibodies to CD45 (BioLegend; San Diego, CA, USA), CD140a (BioLegend) and FluoZin-3-acetoxymethyl (AM) (CD45^−^FluoZin-3-AM^+^; ThermoFisher, Waltham, ME, USA) [22] before being analysed by flow cytometry (LSRII; BD Bioscience, San Diego, CA, USA).

### Quantitative PCR

Pancreatic islets were isolated as described above. RNA from islets of 3–4-week-old female *Tlr9*^*+/+*^ NOD mice and *Tlr9*^−/−^ NOD mice was extracted with an RNAeasy kit (Qiagen, Hilden, Germany) and quantified by NanoDrop (ThermoFisher). Equal amounts of RNA were reverse transcribed using SuperScript III First-strand synthesis kit with random hexamers (Invitrogen, Carlsbad, CA, USA). Quantitative PCR (qPCR) was performed using the Bio-Rad iQ5 qPCR detection system (Hercules, CA, USA) with the specific primers for *Pdx-1* (also known as *Pdx1*) (5′-CAGCAGAACCGGAGGAGAAT-3′ and 5′-CGACGGTTTTGGAACCAGAT-3′) and *Ngn3* (also known as *Neurog3*) (5′-CCCGCAGCTCTCTGTTCTTT-3′ and 5′-GGGTCTCTTGGGACACTTGG-3′) (Sigma). The relative expression of mRNA levels was determined with the 2^−∆∆Ct^ method by normalisation with the housekeeping gene *Gapdh* (5′-AGGTCGGTGTGAACGGATTTG-3′ and 5′-TGTAGACCATGTAGTTGAGGTCA-3′).

### Cell staining for flow cytometry

For direct staining, single-cell suspensions (~5 × 10^4^ to 2 × 10^5^ cells) of immune cells or islet cells were incubated with a 2.4G2 Fc-blocking antibody (10 mins, room temperature) prior to staining with pre-titrated amounts of monoclonal antibodies conjugated with different fluorochromes to combinations of CD3 (17A2), CD4 (GK1.5), CD44 (IM7), CD45 (30-F11) CD62L (MEL-14), CD140a (APA5) and a viability dye (all from BioLegend) in staining buffer (PBS containing 1% FCS) and kept on ice and in the dark for 30 min. The cells were washed twice with 2 ml staining buffer and fixed with 200 μl fixation buffer (eBioScience; San Diego, CA, USA) before analysis by flow cytometry. All antibodies were titrated using mouse splenocytes at different dilutions with the final dilution applied found to be most appropriate for the particular batch of antibody used and our flow cytometer set up.

#### Intracellular staining

For intracellular staining, the single-cell suspension was treated with Perm/Fix buffer (eBioscience) followed by pre-titrated monoclonal antibodies conjugated with different fluorochromes to FoxP3 (FJK-16S, eBioscience) or FluoZin-3-AM (ThermoFisher). After 30 min incubation on ice or at room temperature, the cells were washed twice with 2 ml staining buffer and analysed by flow cytometry. FoxP3 was titrated using mouse splenocytes at different dilutions with the final dilution applied found to be appropriate for the batch used and our flow cytometer set up. For Fluozin-3-AM, mouse islets were used to titrate the antibody, with 1:2000 dilution used found to be appropriate for the particular batch of antibody used and our flow cytometer set up. Dilutions were determined where they gave the clearest separation from the negative background or isotype control.

#### Insulin release assay

An insulin release assay was performed as previously described [23] with modification. Hand-picked pancreatic islets from randomly selected *Tlr9*^−/−^ and *Tlr9*^+/+^ NOD or C57BL/6 mice (5–6 weeks old) were equally distributed to 30 islets/tube after stabilising with low-glucose KRB buffer. The islets were then stimulated with KRB containing high glucose (25 mmol/l) and the supernatant fractions were harvested every 5 min after glucose stimulation. Secreted insulin in the supernatant fractions was measured using the insulin RIA kit (EMD-Millipore, Burlington, ME, USA).

#### Evaluation of islet mass

Ex vivo pancreases from randomly selected 5–6-week-old female *Tlr9*^−/−^ NOD and *Tlr9*^+/+^ NOD mice were fixed in periodate–lysine–paraformaldehyde, sucrose infused and then frozen in Tissue-Tek OCT (Bayer, Elkhart, IN, USA). The pancreas was cut in its entirety into hundreds of 10 μm thick sections and every tenth section was stained with haematoxylin alone (to better visualise the islets) and photographed under the microscope. Islet mass was measured using Image J software (NIH, Bethesda, MD, USA). H&E staining of sections was conducted purely for improving the contrast of the images for the photographs presented in Fig. 4b.

#### In vitro TLR9 antagonist treatment

Freshly isolated islets from *Tlr9*^*+/+*^ NOD mice (5-week-old females) were cultured overnight with the TLR9 antagonist CpG- oligodeoxynucleotides (ODN) (2088; Invivogen, San Diego, CA, USA) or control CpG-ODN (Invivogen), both at 10 μg/ml. After extensive washing, a single-cell suspension was prepared as described earlier and stained with fluorochrome-conjugated monoclonal antibodies to CD45, CD140a and FluoZin-3-AM before analysis by flow cytometry. Another set of freshly isolated islets from *Tlr9*^+/+^ NOD mice was used for insulin release assay, after overnight culture in the presence of the TLR9 antagonist CpG-ODN or control CpG-ODN.

#### In vivo treatment with TLR9 antagonist or chloroquine and diabetes development

Randomly chosen *Tlr9*^*+/+*^ female NOD mice were treated with TLR9 antagonist CpG-ODN (2088) or control ODN, 10 μg/mouse, administered as two i.p. injections, 3 days apart, 1 week after mating. Another set of randomly chosen *Tlr9*^*+/+*^ pregnant female NOD mice were treated with chloroquine (20 μg/g body weight), administered as two i.p. injections, 3 days apart. The female offspring from the treated mothers were investigated for CD140a-expressing islet beta cells, the number of islet beta cells and insulin-secreting function at ~5 weeks old. A third group of randomly chosen pregnant female *Tlr9*^*+/+*^ NOD mice were also treated with antagonist CpG-ODN or control ODN and the natural history of diabetes development was observed in the female progeny of the treated pregnant mice.

#### Statistical analysis

No data were excluded and all viable mice within the different genotypes were included, with the exception of any obvious runts or under-developed mice. No outcomes or conditions were measured or used that are not reported in the results section. Statistical analyses were performed using GraphPad Prism software (San Diego, CA, USA). Diabetes incidence was compared using logrank test. The in vivo and in vitro assays were analysed with Student’s unpaired *t* test or ANOVA for statistical significance.

## Results

### TLR9 deficiency suppressed type 1 diabetes development and enhanced islet beta cell function

**Fig. 1:**
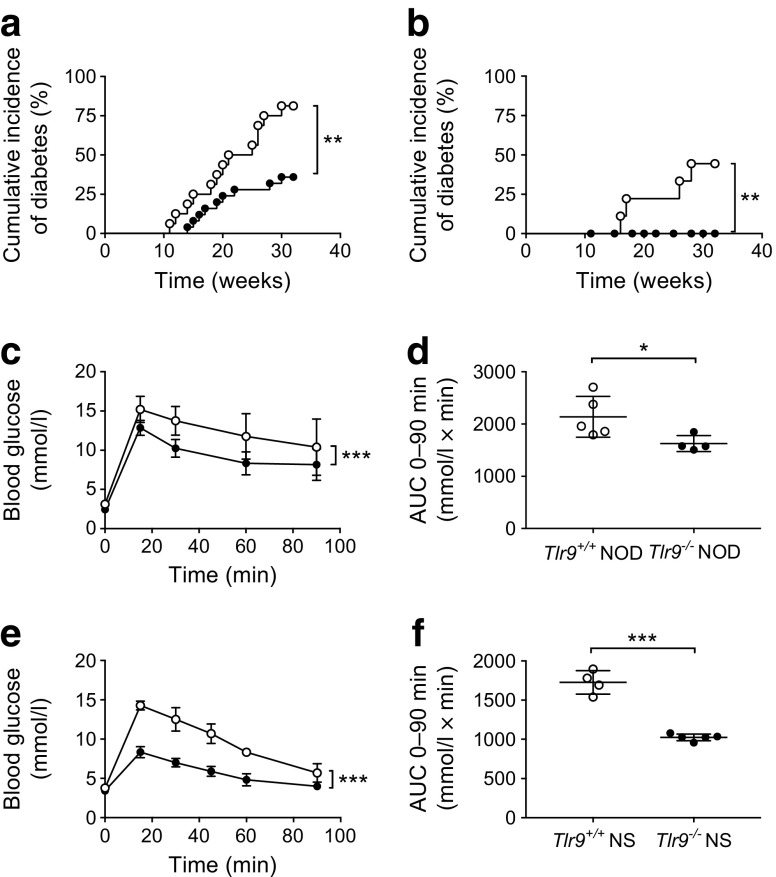
TLR9 deficiency protects NOD mice from diabetes development and enhances beta cell function. (**a**, **b**) The natural history of diabetes development in female (**a**) and male (**b**) *Tlr9*^−/−^ NOD mice (black circles; female, *n* = 25, and male, *n* = 26, mice) and *Tlr9*^+/+^ NOD littermates (white circles; female, *n* = 16, and male, *n* = 9, mice). (**a**) *p* = 0.004 and (**b**) *p* = 0.0025. (**c**, **d**) IPGTTs were performed in 5–6-week-old female *Tlr9*^−/−^ NOD mice (black circles, *n* = 4 mice) and *Tlr9*^+/+^ NOD littermates (white circles, *n* = 5 mice). Blood glucose measurements at different time points after glucose injection (**c**) (*p* = 0.0002) and AUC for glucose (**d**) (*p* = 0.024) are shown. (**e**, **f**) IPGTTs were performed in 5–6-week-old female *Tlr9*^−/−^ NOD mice (*n* = 4 mice) and *Tlr9*^+/+^ NOD.Scid (NSc) mice (*n* = 5 mice). Blood glucose measurements (**e**) and AUC (**f**) are shown. The experiments shown in (**a**) were performed twice with very similar results and one of the two experiments is shown. The experiments shown in (**c**–**f**) were performed three times and the results from one of the three experiments are shown. Data in (**c**–**f**) are expressed as means (SD). Data were analysed in (**a**, **b**) by logrank test for survival, (**c**, **e**) by two-way ANOVA and in (**d**, **f**) by two-tailed unpaired Student’s *t* test. *p < 0.05, **p < 0.01, ***p < 0.001

Although the environment influences type 1 diabetes development [24], particularly in NOD mice, which are very sensitive to environmental changes [25], the protection from diabetes development seen in *Tlr9*^−/−^ NOD mice has been consistent in our mouse colony over many years (Fig. 1a, b). This implies that environmental variation, including housing status (data not shown), does not play a major role in this protection. To test the hypothesis that, beyond its function in innate immunity, TLR9 may impact on pancreatic beta cells and investigate the role of TLR9 in islet beta cell function, we performed glucose tolerance tests (GTTs). *Tlr9*^−/−^ NOD mice had significantly better glucose tolerance, on glucose stimulation in vivo, than their *Tlr9*^*+/+*^ NOD (WT) littermates (Fig. 1c, d), at 5–6 weeks of age, when there is little beta cell destruction in the *Tlr9*^+/+^ NOD mice. To confirm that the improved glucose tolerance in *Tlr9*^−/−^ NOD mice was not due to the reduced insulitis in these mice [10], we generated *Tlr9*^−/−^NOD.Scid mice, which are completely free of lymphocytic infiltration. We tested glucose tolerance in *Tlr9*^−/−^ NOD.Scid mice and *Tlr9*^+/+^ NOD.Scid mice, neither of which develop insulitis nor diabetes. The *Tlr9*^−/−^ NOD.Scid mice demonstrated significantly better glucose tolerance than their *Tlr9*^+/+^ NOD.Scid counterparts (Fig. 1e, f), similar to immune-sufficient *Tlr9*^−/−^ NOD mice.

**Fig. 2:**
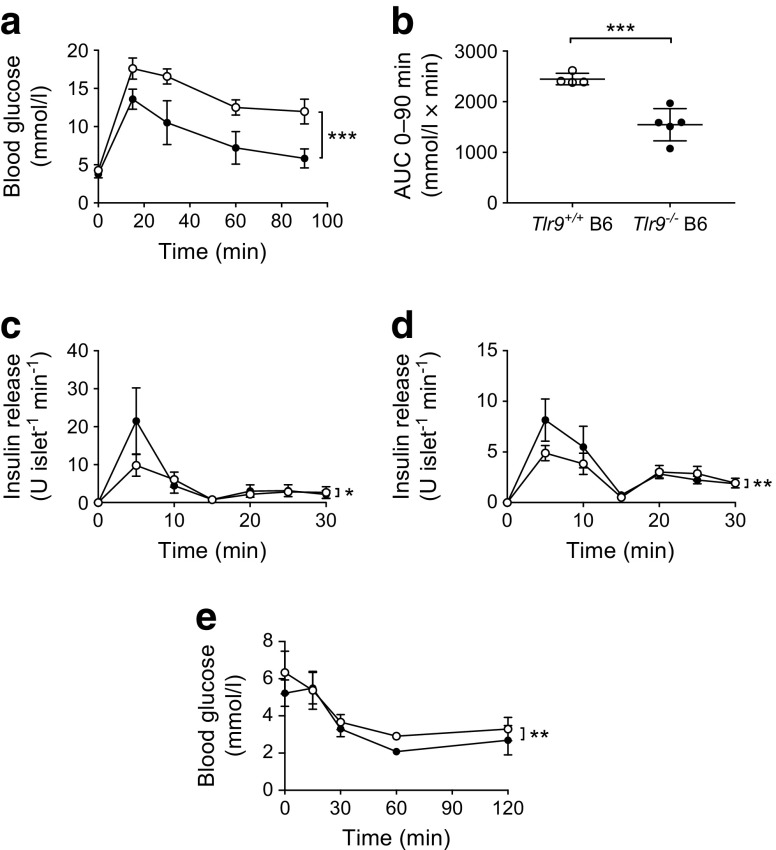
Enhanced beta cell function is not restricted to the NOD mouse strain. (**a**, **b**) IPGTTs were carried out in 5–6-week-old female *Tlr9*^−/−^ (black circles; *n* = 5 mice) and *Tlr9*^+/+^ (white circles; *n* = 4 mice) C57Bl/6 (B6) mice. Blood glucose measurements (**a**) (*p* < 0.001) and AUC for glucose (**b**) (*p* = 0.0006) are shown. (**c**, **d**) An insulin release assay was performed using pancreatic islets from female 5–6-week-old *Tlr9*^−/−^ (black circles) and *Tlr9*^+/+^ (white circles) NOD mice (**c**; *n* = 4 mice) (*p* = 0.038) and C57BL/6 mice (**d**; *n* = 5 mice) (*p* = 0.007); (**e**) ITT in 5–6-week-old female *Tlr9*^−/−^ C57BL/6 (black circles; *n* = 4 mice) and *Tlr9*^+/+^ (white circles; *n* = 4 mice) C57BL/6 mice (*p* = 0.0083). All the experiments were performed twice using *n* = 4 or 5 mice per group per experiment. Data are expressed as means (SD). Data were analysed in (**a**, **c**–**e**) by two-way ANOVA and in (**b**) by two-tailed unpaired Student’s *t* test. *p < 0.05, **p < 0.01, ***p < 0.001

To test whether the improved beta cell function in the absence of TLR9 was related to the NOD genetic background, we studied *Tlr9*^*+/+*^ C57BL/6 mice and *Tlr9*^−/−^ C57BL/6 mice. Interestingly, we consistently found enhanced glucose tolerance in *Tlr9*^−/−^ mice, regardless of their genetic background (Fig. 2a, b). Next, we investigated in vitro insulin secretion of islet beta cells, in response to glucose stimulation. Pancreatic islets isolated from young *Tlr9*^*+/+*^ NOD mice and *Tlr9*^−/−^ NOD mice were cultured in high glucose concentrations and insulin release into the culture supernatant fractions was measured every 5 min. Consistent with the in vivo GTT results, more insulin was secreted from the islets isolated from both *Tlr9*^−/−^ NOD and *Tlr9*^−/−^ C57BL/6 mice vs *Tlr9*^*+/+*^ NOD and C57BL/6 mice (Fig. 2c, d). To further assess the insulin sensitivity, we also conducted an ITT and found improved glucose control in *Tlr9*^−/−^ C57BL/6 mice vs *Tlr9*^*+/+*^ C57BL/6 mice (Fig. 2e).

### Increased islet and beta cell number in the absence of TLR9 in NOD mice

**Fig. 3:**
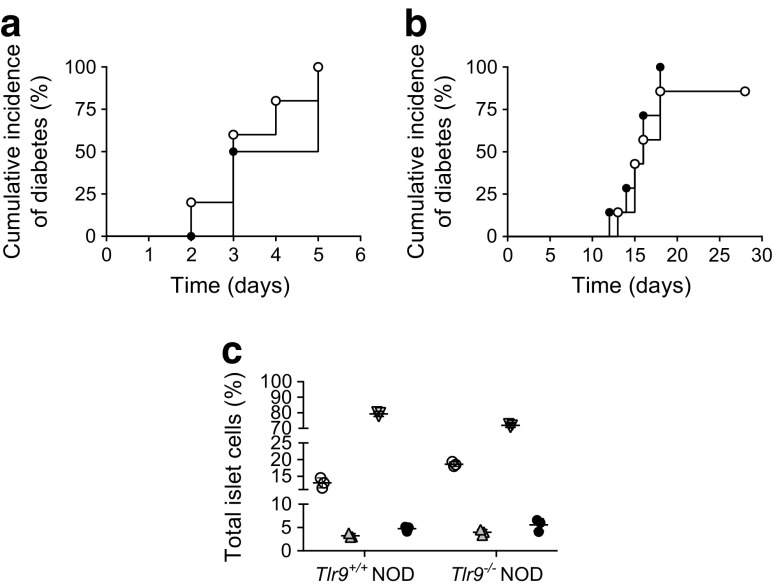
TLR9-deficiency does not protect from beta cell death induced by STZ. (**a**) Incidence of diabetes development induced by high-dose STZ (100 mg/kg, two i.p. injections) in 5–6-week-old female *Tlr9*^−/−^ NOD mice (black circles; *n* = 5 mice) and *Tlr9*^+/+^ NOD littermates (white circles; *n* = 5 mice). (**b**) Incidence of diabetes development induced by low-dose STZ (40 mg/kg, five i.p. injections) in 5–6-week-old female *Tlr9*^−/−^ NOD mice (black circles; *n* = 7 mice) and *Tlr9*^+/+^ NOD littermates (white circles; *n* = 7 mice). (**c**) Apoptosis of islet cells in response to STZ (40 ng/ml) insult in vitro determined by Annexin V and 7-AAD staining (*n* = 4 mice per group). White circles, live cells (Annexin V^−^/7-AAD^−^); grey triangles, early apoptotic cells (Annexin V^+^/7-AAD^−^); inverted white triangles, late apoptotic cells (Annexin V^−^/7-AAD^+^); black circles, dead cells (Annexin V^+^/7-AAD^+^). All experiments were performed once. Data in (**c**) are expressed as means (SD). Data in (**a**, **b**) were analysed by logrank test for survival and in (**c**) by two-tailed unpaired Student’s *t* test (NS for all)

Based on our findings of enhanced beta cell function, we hypothesised that, in the absence of TLR9, beta cells were either more resistant to cell death or had increased cell growth. To investigate beta cell death, we treated *Tlr9*^*+/+*^ NOD and *Tlr9*^−/−^ NOD mice with STZ, a chemical causing beta cell death leading to clinical diabetes. However, there was no particular resistance to beta cell death in *Tlr9*^−/−^ NOD mice, as diabetes onset was similar to the onset in their *Tlr9*^*+/+*^ NOD counterparts after STZ treatment, both at high dose and multiple low doses (Fig. 3a, b). We also investigated islet apoptosis in response to STZ, directly ex vivo. Freshly isolated islet cells from *Tlr9*^*+/+*^ NOD and *Tlr9*^−/−^ NOD mice were cultured with STZ (40 ng/ml) for 3 h followed by staining for apoptosis using Annexin V and 7-AAD. In line with the results from the in vivo experiments, islet cells from *Tlr9*^−/−^ NOD mice were as susceptible to apoptosis as the islet cells from *Tlr9*^+/+^ NOD mice (Fig. 3c).

**Fig. 4:**
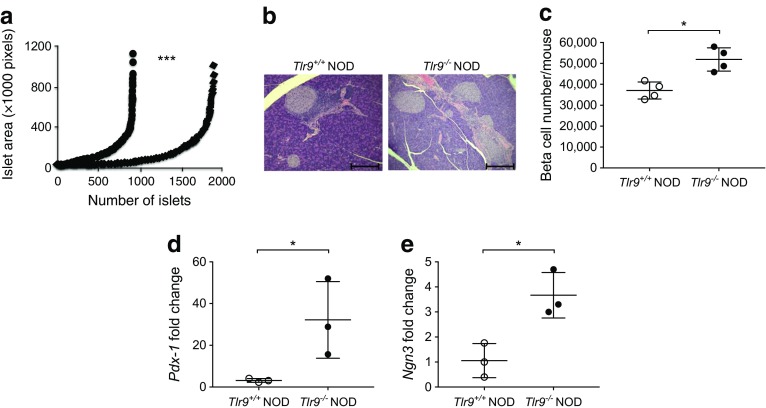
TLR9-deficiency promotes beta cell development. (**a**) Photographs were taken of pancreatic sections after staining with haematoxylin alone to visualise the islets better. Islet area was evaluated by ImageJ software. More islets were present in the pancreases of *Tlr9*^−/−^ NOD mice (black diamonds) compared with *Tlr9*^+/+^ NOD mice (black circles) (*p* < 0.001). (**b**) Representative pancreas sections after staining with H&E are shown. Scale bar, 100 μm. (**c**) Beta cells from pancreatic islets of 4-week-old female *Tlr9*^*+/+*^ NOD and *Tlr9*^−/−^ NOD mice (*n* = 4 mice for both groups) were harvested after treatment with Cell Dissociation Solution (Sigma). After staining with anti-CD45 and FluoZin-AM, beta cells (CD45^−^FluoZin^+^ cells) were enumerated by flow cytometry. Beta cell number was increased in the pancreas of *Tlr9*^−/−^ NOD mice when compared with *Tlr9*^+/+^ NOD mice. (**d**, **e**) Total cellular RNA was extracted from islets of ~4-week-old female *Tlr9*^*+/+*^ NOD and *Tlr9*^−/−^ NOD mice and qPCR was conducted with specific primers for transcription factors for beta cell development, *Pdx-1* (**d**) and *Ngn3* (**e**). The relative expression level of mRNA was determined by normalisation with the housekeeping gene, *Gapdh*. The expression of both *Pdx-1* and *Ngn3* was increased in pancreatic islets of *Tlr9*^−/−^ NOD mice when compared with *Tlr9*^+/+^ NOD mice (*p* = 0.01). The experiments shown in (**c**–**e**) were performed twice, with results from one of the experiments being shown. Data in (**c**–**e**) are expressed as means (SD). Data were analysed in (**a**) by two-way ANOVA and in (**c**–**e**) by two-tailed unpaired Student’s *t* test. *p < 0.05, ***p < 0.001

Next, we investigated the alternative possibility of increased cell growth by analysing the islet mass. OCT-embedded frozen pancreatic tissue blocks from young *Tlr9*^*+/+*^ NOD and *Tlr9*^−/−^ NOD mice were completely sectioned at 10 μm/section and stained with haematoxylin alone to better visualise the islets. We examined every tenth section under light microscopy and measured the islet area. The data were analysed with ImageJ software. Interestingly, we discovered a significant increase in islet mass of the pancreases from *Tlr9*^−/−^ NOD mice compared with their *Tlr9*^*+/+*^ NOD counterparts (Fig. 4a). The presence of more islets in *Tlr9*^−/−^ vs *Tlr9*^+/+^ NOD mouse pancreases (Fig. 4b) contributed to the increased islet mass observed in *Tlr9*^−/−^ NOD mice as examined by H&E staining. We also examined the number of beta cells per pancreas by flow cytometry. Consistent with the islet mass results, the number of beta cells per mouse was much higher in *Tlr9*^−/−^ NOD mice than in *Tlr9*^*+/+*^ NOD mice (Fig. 4c). To confirm the association of TLR9 deficiency with islet beta cell development, we examined the expression of genes encoding pancreatic and duodenal homeobox-1 (PDX-1) and neurogenin 3 (NGN3), two transcription factors known to play an important role in islet beta cell development [26–28]. We compared the expression levels of *Pdx-1* and *Ngn3* mRNA in dispersed islet cells (5 × 10^5^) of freshly hand-picked islets from the pancreases of young WT and *Tlr9*^−/−^ NOD mice, by qPCR. As predicted, the expression of *Pdx-1* and *Ngn3* was significantly higher in islet cells from *Tlr9*^−/−^ NOD mice compared with *Tlr9*^+/+^ NOD mice (Fig. 4d, e). Taken together, our data suggested that TLR9 negatively regulates islet beta cell development and, in the absence of TLR9, islet mass is increased, which leads to enhanced insulin secretion and islet beta cell function.

### Increased islet beta cells expressing CD140a in the absence of TLR9 in NOD mice

**Fig. 5:**
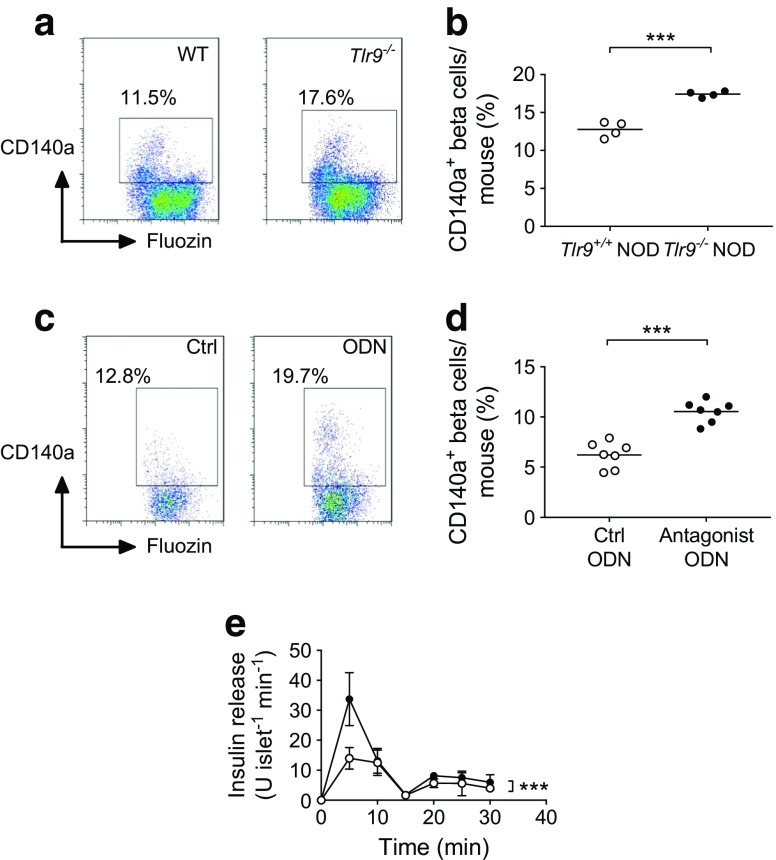
CD140a is highly expressed in *Tlr9*^−/−^ islets. (**a**, **b**) CD140a expression on islet beta cells from *Tlr9*^*+/+*^ NOD and *Tlr9*^−/−^ NOD mice. Pancreatic islets were isolated from ~4-week-old female *Tlr9*^*+/+*^ NOD and *Tlr9*^−/−^NOD mice and beta cells were harvested after treatment with Cell Dissociation Solution (Sigma). The cells were stained with monoclonal antibodies to CD45, CD140a and FluoZin-AM and analysed by flow cytometry. CD45^−^ cells (non-haematopoietic cells) were gated and the expression of CD140a on FluoZin^+^ beta cells is shown (**a**). Quantification of CD140a^+^ beta cells in *Tlr9*^*+/+*^ NOD and *TLR9*^−/−^ NOD mice (**b**) (*p* = 0.0002; *n* = 4 mice [pooled into two samples] per group per experiment), with two experiments being performed). (**c**, **d**) CD140a expression on islet beta cells from *Tlr9*^*+/+*^ NOD mice treated with control (Ctrl) ODN or antagonist ODN. Isolated islets from *Tlr9*^*+/+*^ NOD mice were cultured with TLR9 antagonist ODN or control ODN (both at 10 μg/ml) overnight. Islet beta cells were harvested after dissociation and stained with monoclonal antibodies to CD45, CD140a and FluoZin-AM. CD140a expression is shown on FluoZin^+^ beta cells after gating on CD45^−^ cells (**c**). Quantification of CD140a^+^ beta cells after treatment with TLR9 antagonist ODN or control ODN (**d**) (*p* < 0.001; *n* = 7 mice per group per experiment; data are from two experiments). (**e**) Insulin release assay of islets treated with control ODN (white circles; *n* = 7 mice) or antagonist ODN (black circles; *n* = 7 mice) (*p* < 0.001). Two-tailed unpaired Student’s *t* test was used for (**a**–**d**) and two-way ANOVA was used for (**e**). Data in (**b**) and (**d**) are expressed as the actual numbers, with the mean indicated as the line in the figures. Data in (**e**) are expressed as means (SD). ***p < 0.001

We next investigated the molecular pathway(s) by which TLR9 could influence pancreatic islet development. We focused on CD140a (also known as platelet-derived-growth-factor receptor-α [PDGFRα]), as it has been reported to control proliferation of pancreatic beta cells [29]. We examined CD140a expression on dispersed beta cells from *Tlr9*^*+/+*^ NOD and *Tlr9*^−/−^NOD mice, using anti-mouse-CD140a and the beta cell marker FluoZin-AM [30], and analysed the cells by flow cytometry. FluoZin-AM is a zinc-selective fluoroionophore that can detect the high concentration of zinc present in the insulin granules within beta cells [31]. We found that more beta cells from *Tlr9*^−/−^ NOD mice expressed CD140a than their *Tlr9*^*+/+*^ NOD counterparts (Fig. 5a, b). Based on these results, we hypothesised that inhibition of TLR9 signalling in *Tlr9*^*+/+*^NOD mice may induce CD140a expression on beta cells and enhance their function. To test our hypothesis, we cultured the islets from *Tlr9*^*+/+*^ NOD mice with the TLR9 antagonist CpG-ODN 2088 or control CpG-ODN overnight and examined CD140a expression. It is interesting that the TLR9 antagonist was indeed able to induce CD140a expression on *Tlr9*^*+/+*^ islets (Fig. 5c, d). Next, we tested the function of beta cells from TLR9 antagonist-treated WT islets by measuring insulin secretion in response to glucose stimulation. Pancreatic islets isolated from young *Tlr9*^*+/+*^ NOD mice were cultured with TLR9 antagonist ODN or control ODN overnight. After extensive washing, the islets were further cultured in high glucose concentrations and insulin release into the culture supernatant fractions was measured every 5 min. Figure 5e shows that early-phase insulin secretion in response to glucose stimulation was significantly enhanced in *Tlr9*^*+/+*^ NOD islets treated with TLR9 antagonist. To test whether inhibition of CD140a could reverse the effect of TLR9 antagonist on beta cells, we cultured islets from young *Tlr9*^*+/+*^ NOD and *Tlr9*^−/−^ NOD mice with a CD140a inhibitor (PDGFR inhibitor, Enzo Life Science, Oyster Bay, NY, USA), as we had done with the TLR9 antagonist. However, due to the cell toxicity of the inhibitor, most of the islet cells were not viable after overnight culture (data not shown).

### Treatment with TLR9 antagonist resulted in increased CD140a-expressing islet beta cells and number of beta cells, improved beta cell function and protected *Tlr9*^*+/+*^ NOD mice from diabetes development

**Fig. 6:**
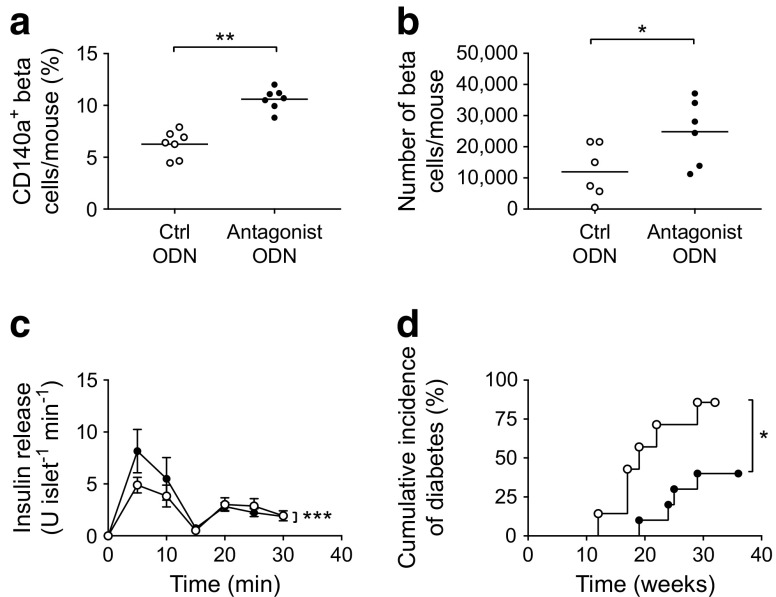
Treatment of *Tlr9*^*+/+*^ NOD mice with TLR9 antagonist recapitulated *Tlr9*^−/−^ phenotypes in vivo. (**a**) Treatment with TLR9 antagonist enhances CD140a expression on islet beta cells from *Tlr9*^*+/+*^ NOD mice. CD140a expression on islet beta cells from the female progeny (5–6 weeks old) of TLR9 antagonist ODN- or control (Ctrl) ODN-treated pregnant NOD mice (*p* = 0.0095; *n* = 7 mice for both groups). (**b**) TLR9 antagonist treatment increases beta cell number. Number of beta cells/mouse in the female progeny (5–6 weeks old) of TLR9 antagonist ODN- or Ctrl ODN-treated pregnant NOD mice (*p* = 0.044; *n* = 6 mice for both groups). (**c**) TLR9 antagonist treatment enhances beta cell function. Insulin release from islets from the female progeny (5–6 weeks old) of TLR9 antagonist ODN- (black circles) or Ctrl ODN-treated (white circles) pregnant NOD mice (*p* = 0.0007; *n* = 6 mice for both groups). (**d**) TLR9 antagonist treatment prevents spontaneous type 1 diabetes development. Pregnant NOD mice (*n* = 3) were treated with TLR9 antagonist ODN and the incidence of diabetes was monitored in the female progeny (*p* = 0.019) (black circles; *n* = 10 mice) or Ctrl ODN (white circles, *n* = *7* mice). Two independent experiments were performed in (**a**–**c**), *n* = 3–4 mice per group per experiment. Data were analysed in (**a**, **b**) by two-tailed unpaired Student’s *t* test, in (**c**) by two-way ANOVA and in (**d**) by logrank test for survival. Data are expressed in (**a**) and (**b**) as means (horizontal line) and in (**c**) as means (SD). *p < 0.05, **p < 0.01, ***p < 0.001

**Fig. 7:**
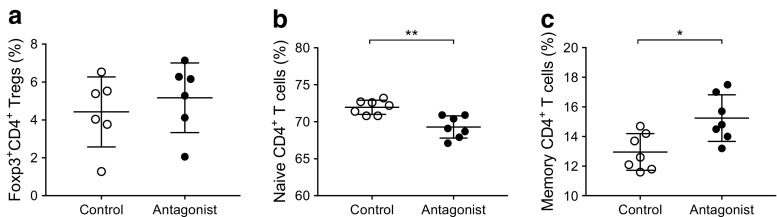
Effect of TLR9 antagonist treatment on immune cells in *Tlr9*^*+/+*^ NOD mice. (**a**) Regulatory T cells (Foxp3^+^ Tregs) in pancreatic islets. Immune cells were extracted from pancreatic islets isolated from the female progeny (5–6 weeks old) of *Tlr9*^*+/+*^ NOD mice treated with TLR9 antagonist ODN or control ODN during pregnancy (*n* = 3 pregnant dams/group). Cells were stained with monoclonal antibodies to CD3, CD4 and Foxp3. The percentage of Foxp3^+^CD4 T cells is shown after gating for CD3^+^ cells (*p* = 0.235; *n* = 6 mice for both groups). (**b**) Reduced naive (CD44^−^CD62L^+^) CD4^+^ T cells in the spleen of the female progeny (5–6-weeks of age) from TLR9 antagonist-treated pregnant NOD mice. Splenocytes from these mice were isolated and stained with monoclonal antibodies to CD3, CD4, CD44 and CD62L. The percentage of naive CD4^+^ T cells is shown after gating for CD3^+^CD4^+^ T cells (*p* = 0.0011; *n* = 7 mice for both groups). (**c**) Increased memory (CD44^+^CD62L^−^) CD4^+^ T cells in the spleen of the female progeny (5–6 weeks old) from TLR9 antagonist-treated pregnant NOD mice. Splenocytes from these mice were isolated and stained with monoclonal antibodies to CD3, CD4, CD44 and CD62L. The percentage of memory CD4^+^ T cells is shown after gating for CD3^+^CD4^+^ T cells (*p* = 0.0106; *n* = 7 mice for both groups). Two independent experiments were carried out. Data are expressed as means (SD). Data were analysed by two-tailed unpaired Student’s *t* test. *p < 0.05, **p < 0.01

**Fig. 8:**
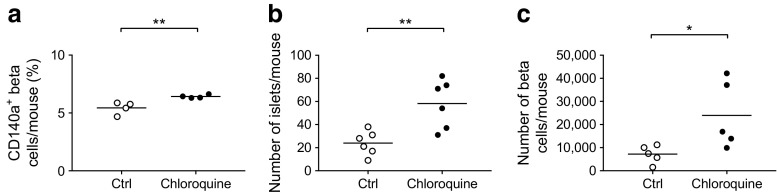
The TLR9 inhibitor chloroquine promotes islet beta cell development. Pregnant *Tlr9*^*+/+*^ NOD mice were treated with chloroquine (20 μg/g body weight, i.p. injection, twice, 3 or 4 days apart) or PBS (control). (**a**) Chloroquine treatment increased CD140a^+^ beta cell per cent. Islet beta cells from 4-week-old female progeny of chloroquine-treated (black circles) or PBS-treated (white circles) pregnant *Tlr9*^*+/+*^ NOD mice (*n* = 3 pregnant dams) were examined by flow cytometry for CD140a expression (*p* = 0.0017; *n* = 4 mice for both; pancreatic islets from two mice were pooled as one sample). (**b**) Chloroquine treatment increased the number of islets. Pancreatic islets were isolated and counted from 4-week-old female progeny of chloroquine-treated (black circles) or PBS-treated (white circles) mothers (*p* = 0.005; *n* = 6 for both). (**c**) Chloroquine treatment increased the number of beta cells. Islet beta cells were isolated and stained with monoclonal antibodies to CD45 and FluoZin-AM. The number of beta cells (CD45^−^Fluozin-AM^+^) was counted by flow cytometry (*p* = 0.0188; *n* = 5 mice for both). Two independent experiments were performed, with *n* = 4–6 mice per group per experiment. Data are expressed as means (horizontal line). Data were analysed by two-tailed unpaired Student’s *t* test. *p < 0.05, **p < 0.01

A study by Chen and colleagues suggested that the expression of CD140a on islet beta cells was age-dependent [29]. We hypothesised that the enhanced development of pancreatic islets and the increased number of CD140a-expressing beta cells in *Tlr9*^−/−^ NOD mice occur early in life. To test this in vivo, we treated *Tlr9*^*+/+*^ NOD mice with TLR9 antagonist CpG-ODN and its control CpG-ODN prenatally (i.e. during fetal development). We injected female *Tlr9*^*+/+*^ NOD mice, 1 week after mating, with CpG-ODN 2088 or control CpG-ODN (10 μg/mouse, i.p., twice a week for 2 weeks) and examined the numbers of beta cells and CD140a-expressing beta cells from the young female progeny (5 weeks old). No adverse effects were observed in mice receiving this treatment. In line with our findings in *Tlr9*^−/−^ NOD mice, we demonstrated an increase in the number of CD140a-expressing islet beta cells, as well as the number of islets and beta cells per mouse, in the mice that received TLR9 antagonist ODN during embryonic development (Fig. 6a, b). To test whether the function of beta cells was also improved, we performed insulin release assays. Consistent with the results found in *Tlr9*^−/−^ NOD mice, TLR9 antagonist treatment in *Tlr9*^*+/+*^ NOD mice also enhanced beta cell function, as demonstrated by the secretion of significantly more insulin in response to glucose stimulation (Fig. 6c). Furthermore, the offspring of the *Tlr9*^*+/+*^ NOD mice treated with TLR9 antagonist CpG-ODN had significantly delayed and overall lower incidence of diabetes than the progeny of the mice treated with control CpG-ODN (Fig. 6d). We have previously shown that TLR9 deficiency increases the expression levels of CD73, which is also expressed on regulatory T cells, and that TLR9-deficient mice are protected from diabetes [10]. Given the finding that islet beta cell number and function are increased by TLR9 antagonist treatment, we examined immune variables in the mice treated with TLR9 antagonist. We found that the percentage of regulatory T cells was increased in the islets of the mice whose mothers had received TLR9 antagonist ODN during pregnancy, although the increase was not statistically significant (Fig. 7a). Interestingly, the naive and memory CD4^+^ T cells in the spleen of these mice showed significant changes when compared with the progeny of control ODN-treated mice (Fig. 7b, c). It is known that chloroquine acts as a TLR9 inhibitor. Therefore, to test whether chloroquine could also promote islet and beta cell development, we prenatally treated pregnant *Tlr9*^*+/+*^ NOD mice with chloroquine (i.p. injection, 20 μg/g body weight, twice with a 3 day interval) and assessed the number of islets and beta cells from the female progeny at ~5 weeks of age. It is intriguing that chloroquine had very similar effects to the TLR9 antagonist ODN on the development of islet beta cells, including increasing the proportion of CD140a-expressing islet beta cells (Fig. 8a) and increasing the numbers of islets and beta cells (Fig. 8b, c). Thus, our results suggest that blocking TLR9 signalling promotes islet beta cell development.

## Discussion

In this study, we have identified a novel function of TLR9, quite distinct from its role in innate immunity. We showed that TLR9-deficient mice have more pancreatic islets and, correspondingly, more islet beta cells, with increased glucose-stimulated insulin secretion in vitro and improved glucose tolerance in vivo. This was not due to increased resistance to beta cell death. Rather, we found increased expression of genes encoding PDX-1 and NGN3, transcription factors associated with beta cell development, suggesting that the increase in beta cell mass was related to promotion of beta cell growth. Although many growth factors regulate islet beta cell development [28, 32], in linking TLR9 deficiency and islet beta cell development, we found that the proportion of islet beta cells expressing CD140a was increased in TLR9-deficient mice. Confirming that this effect was associated with TLR9 deficiency, we showed, using TLR9 antagonism, that inhibition of the TLR9 signalling pathway in islets from TLR9-sufficient mice led to an increased number of CD140a-expressing beta cells and enhanced insulin secretion in response to glucose stimulation. Our results thus demonstrate a novel link between TLR9 and CD140a, a growth factor that has been reported to regulate islet beta cell proliferation [29].

Islet beta cells display distinct phases of significant growth in the fetal and neonatal periods, whereas there is little increase in islet beta cell numbers in adulthood in either mice or humans [33, 34]. Proliferation and survival are among the functions promoted by platelet-derived growth factor (PDGF) signalling through the PDGF receptors, of which CD140a is one of two main receptor isoforms for PDGF. This is a receptor tyrosine kinase and it is expressed in cells of mesenchymal origin, including the pancreas [35]. It has been suggested that the human CD140a promoter has a binding site for c-Rel [36], which is a subunit of the NFκB protein complex and plays an important role in development, immunity and diseases, including type 1 diabetes [37–39]. Expression of CD140a is normally age-dependent in mouse pancreatic islet beta cells, reaching a peak at around the age of 2 weeks and declining once mice reach adulthood [29]. If this receptor is lost prematurely, as shown by gene mutation experiments, beta cell proliferation and expansion are impaired [29]. Our current results suggest that TLR9 may be one of the factors involved in the control of the age-dependent expression of CD140a, and may negatively regulate the expression of CD140a. Blocking TLR9 signalling, either by genetic targeting or by inhibition via small molecules early in life, results in an increase in CD140a expression and a corresponding increase in islet beta cells later in life.

There is increasing evidence to suggest that TLRs not only recognise exogenous ligands from microbes but also recognise endogenous ligands from both normal and damaged cells. As TLR9 recognises CpG motifs, present in bacterial DNA or endogenous DNA, that are released from both physiological and pathological dying cells [40], it could play a role in tissue remodelling. Although the gut microbiota differs in composition when comparing *Tlr9*^−/−^ NOD and *Tlr9*^+/+^ NOD mice, protection from diabetes and enhanced beta cell development and function were not associated with this difference (data not shown). It is particularly interesting that TLR9 is linked to expression of a growth factor receptor, signalling through which affects growth and development in a tissue-specific manner. Islet beta cells undergo significant growth and remodelling during prenatal development [41] but the capacity for neogenesis and regeneration of beta cells is lost later in life. Marked beta cell hyperplasia occurs during neonatal development and the role of CD140a in beta cell proliferation is age-dependent [29]. In our experiments, brief treatment of pregnant *Tlr9*^+/+^ NOD mice with a TLR9 antagonist oligonucleotide, as well as with chloroquine (which also antagonises TLR9), significantly enhanced beta cell growth. This was accompanied by enhanced insulin secretion in response to glucose stimulation, as the mice developed into adulthood, and also coincided with an increased percentage of CD140a-expressing beta cells, suggesting the association of the two processes. We did not examine the effect of chloroquine on islet beta cells directly in vitro, as we think it is important to study the drug effect in vivo; although chloroquine is not specific for islet beta cells, we did not observe any noticeable systemic adverse effects.

TLR signalling in mammals has been mainly linked to innate immunity. Our results suggest a novel effect of TLR9 on growth and development in addition to its role in innate immunity. The fact that this brief inhibition of TLR9 signalling, early in life, led to protection from autoimmune diabetes development in *Tlr9*^+/+^ NOD mice, similar to the phenotype seen in *Tlr9*^−/−^ NOD mice, suggests a possible means of improving islet reserve. The inhibition of the development of diabetes is likely to be a combination of increased beta cell capacity (referring to increase in number of beta cells, improved sensitivity to glucose and increased insulin production), together with the immunological changes that we, and others, have previously reported [9, 10]. These changes include increased expression of CD73 and reduced production of proinflammatory cytokines [10], together with a reduction in activation of autoreactive diabetogenic CD8 T cells, all of which occur as a result of TLR9 inhibition [9]. Inhibition of TLR9 has not been explored in human type 1 diabetes; however, pre-clinical tests for identifying an effective and safe dose, route and timing of any potential agent would need to be conducted first.

We conclude that TLR9 negatively regulates the development of pancreatic islets and insulin-secreting beta cells, mediated, at least in part, by CD140a. Our findings provide novel insight into the function of TLR9 beyond the immune cells and also suggest a new direction for the design of preventive and/or therapeutic strategies for diabetes.

## Data Availability

Data are available on request from the authors.

## Abbreviations

AM: Acetoxymethyl
IPGTT: Intra-peritoneal glucose tolerance test
ITT: Insulin tolerance test
NGN3: Neurogenin 3
ODN: Oligodeoxynucleotides
PDGF: Platelet-derived growth factor
PDGFRα: Platelet-derived growth factor receptor-α
PDX-1: Pancreatic and duodenal homeobox-1
qPCR: Quantitative PCR
STZ: Streptozotocin
TLR: Toll-like receptor
WT: Wild-type
